# Supplementary material for: Indigenous knowledge of Rift Valley Fever among Somali nomadic pastoralists and its implications on public health delivery approaches in Ijara sub-County, North Eastern Kenya
Source: PLoS Negl Trop Dis. 2021 Feb 22;15(2):e0009166. doi: 10.1371/journal.pntd.0009166 (PMC7932528; doi:10.1371/journal.pntd.0009166)
Supplement: Attachment — Submitted filename: Review of the Manuscript (PNTD-D-19-02196).docx [file pntd.0009166.s001.docx]

Review of the Manuscript Number PNTD-D-19-02196

**Introduction**

The manuscript titled *indigenous knowledge of Rift Valley Fever among Somali nomadic pastoralists and its implication on public health delivery approaches in Ijara Division, North Eastern Kenya* presents important anthropological information that can be utilized by authorities immediately before and during outbreaks of Rift Valley Fever (RVF). Specifically, the manuscript mentions aspects of disease knowledge from an emic perspective including perceived risk factors associated with the disease including heavy rainfall, swarms of mosquitoes, biting flies and contact with wild animals. Symptoms including froth from nose, bloody diarrhoea, fever, abortions and salivation. The manuscript further points out risk pathways associated with RVF including mosquito bites, drinking raw animal milk and/or blood, infected livestock meat and infected wild animals. The authors postulate that the above anthropological knowledge may be useful in the development of early warning system and the general disease control mechanisms when outbreaks occur.

The information is important to officers working in the domain of public health and medical anthropology in general.

However, there are sections that the authors need to review before publication. These include:

**Topic**

i. The topic appears lengthy. It is 24 words long. Should revise to at most 16 words.

**Abstract**

ii. The abstract should be continuous without sections.
iii. The authors ought to know that a zoonotic disease is a disease that affects both humans and animals hence avoid repetition i.e. use either
iv. The abstract does not have study methods but research design

**Author Summary**

v. Blood is also a body fluid hence repetition as in the manuscript
vi. The mosquito is the vector of the disease. Categorization of the mosquito as secondary vector is therefore confusing. The Aedes and Culex mosquitoes are the vectors for RVF ( See WHO 2018- RVF key facts).
vii. Results have been presented in the Introduction section (Page 6). Sections should have appropriate information

**Methods**

viii. The manuscript presents Provinces. The Manuscript is not live to contemporary administrative divisions in Kenya i.e. Counties.
ix. The activity was carried out among the Somali pastoralists who constituted 90% of the research site. There is no account of the remaining 10% non-Somali inhabitants.
x. There are two sample size determination approaches. The Cochran determination appears irrelevant.

**Results and Discussion**

xi. The labels of figures and tables should be inserted appropriately and also their inclusion in text should be avoided
xii. There is need for consistency in recording of perceived symptoms e.g. in some instances it indicates bloody nose and in some froth from nose.
xiii. When having verbatim quotes, there is need to include source of the quote and place of acquisition. The source should be placed appropriately below the quotes
xiv. The structure of in-text reference should be revised i.e. the sources to appear in numbers and appropriate consistency maintained
xv. All non-English terms should be italised .
xvi. In Gender role matrix the authors should be cognizant of the fact that milking camels among the Somali is a male function

**Conclusion**

xvii. *Retrogressive* cultural practice is not an anthropological conclusion.
