# Supplementary material for: Indigenous knowledge of Rift Valley Fever among Somali nomadic pastoralists and its implications on public health delivery approaches in Ijara sub-County, North Eastern Kenya
Source: PLoS Negl Trop Dis. 2021 Feb 22;15(2):e0009166. doi: 10.1371/journal.pntd.0009166 (PMC7932528; doi:10.1371/journal.pntd.0009166)
Supplement: Attachment — Submitted filename: Author Letter on Responses to Reviewers_June4_2020.docx [file pntd.0009166.s002.docx]

Dr. Geoffrey Otieno Muga, PhD

Corresponding and First Author

Institute of Anthropology Gender and African Studies

University of Nairobi

P. O. Box 30197

Nairobi

June 4, 2020.

The Editorial Manager

PLOS Neglected Tropical Diseases

Dear Sir/Madam,

**Re: Submission of Revised Manuscript: Indigenous knowledge of Rift Valley Fever among Somali pastoralists and its implications on public health delivery approaches in Ijara sub-county North Eastern Kenya**

I take this opportunity on behalf of the authors to thank you very much for sharing with us reviewers’ comments on our manuscript cited above. We have made great efforts to address all the comments by the 3 reviewers. We found the comments very useful and they have made us to greatly enrich the manuscript and make it more comprehensive. Find below for your review our responses to the reviewers’ comments and a description of changes made in each and every section.

**Section: topic, abstract, author summary and introduction**

**Reviewer # 1:**

- The topic has been left intact as also suggested by the editorial team.
- The abstract section has been made continuous by deleting the sub-sections
- Repetition caused by referring to RFV as a zoonotic disease that affects both humans and animals has been corrected by rephrasing the first statement on abstract section.
- Study methods have been added to the abstract section.
- Repetition of using blood and body fluid has been corrected by deleting the term blood from the author summary section.
- Confusion caused by categorization of mosquitoes as secondary vectors has been corrected by rephrasing the statement in the introduction section. Reference to *culex* and *anopheles* mosquitoes as secondary vectors has been deleted.
- The results that appeared in the introduction section have been deleted and the section re-written for clarity.

**Reviewer # 2: No comments on the section**

**Reviewer # 3: No comments on the section**

**Section: Method**

**Reviewer # 1**

- The new administrative boundaries have been used in the manuscript to reflect counties. The topic has been re-written to capture Ijara sub-county and a map of study site has been added showing Ijara location and the neighbouring counties and Somalia. The study location/site sub-section under methods has also been re-written to reflect the new county boundaries. The whole document has been reviewed and new county boundaries used to replace old boundaries that focussed on districts and divisions.
- The remaining 10% of non-Somali inhabitants have been accounted for in the manuscript.
- Cochran formula has been deleted and Yamane formula upheld.

**Reviewer # 2**

- The comments by the reviewer approved the section as presented. There were no concerns requiring authors’ response.

**Reviewer # 3**

- A table on the demographic characteristics of respondents has been added to the results and discussion section. The data on the table has been adequately discussed.
- A statement on enumeration area in the sample population and sampling procedure sub-section has been rephrased for clarity of the sampling procedure.
- Inclusion/exclusion criteria has been specified under sampling population and sampling procedure
- Cochran formula has been deleted and Yamane formula maintained.
- Elaboration on how secondary review data was used in the study has been done.
- Data management section has been made more concise and comprehensive by detailing how qualitative and quantitative data was managed and analysed in the study.

**Section: Results and Discussion**

**Reviewer #1**

- The labels for figures and tables have been appropriately inserted. They have also been deleted from the text.
- The symptom ‘froth from the mouth’ has been consistently used in the manuscript.
- The source and place of verbatim quote acquisition have been inserted under the quotes.
- The structure of in-text reference has been revised and consistently captured in numbers in the manuscript.
- All non-English terms in the manuscript have been italicised
- In the gender role matrix, the role for milking cattle has been appropriately specified as belonging to women as opposed to milking generally to avoid confusion.

**Reviewer # 2**

- The results and discussion section has been beefed up with more information and analysis to enable drawing conclusions for validating the findings
- A map of Ijara sub-county-the study site has been included in the manuscript.
- The sub-section on RVF entry pathways has been combined with food preparations and consumption practices since they are related. They have been discussed together to make the sub-section more tight and coherent.

**Reviewer # 3**

- The results and discussion section remains combined. PLOS manuscript organization instructions also allow authors flexibility to combine the section into one.
- A sub-section and table of socio-demographic characteristics of respondents has been added into the text together with their discussions.
- The research design sub-section has been strengthened by adding support information stating that the data collected from survey provided a basis for sharpening the qualitative tools that guided the second phase of data collection.

**Section: Conclusion**

**Reviewer #1**

- The word retrogressive has been deleted.

**Reviewer # 2**

- To provide a basis for a more comprehensive conclusion, the whole of the results and discussion section has been revised and more data and information added.
- Based on the data, practical strategies of integrating the knowledge of the nomads into the early warning system have been suggested. These include developing health messages that are well targeted and contextually appropriate given the low level of education of the pastoralists and the difficult-to-reach settlements. Such messages should be developed and disseminated using the local language and popular communication channels such as the religious leaders and local radio stations. Training of the nomads on reporting mechanisms during outbreaks and empowering a selected number of them who own mobile phone devices to report to the authorities any observable changes in livestock and environment are also given as practical strategies to consider.

**Reviewer #3**

- As described in the last bullet above, the conclusion section has been beefed up with details on strategies on how to integrate the knowledge of the pastoralists into an early warning system.
- The gender findings in the conclusion section have been infused with other statements in the section and rephrased to make the conclusion section a more encompassing summary statement.
- The strengths and limitations of the study have been added into the manuscript under method section.

**Editorial and data presentation modifications**

**Reviewer #1: No comment**

**Reviewer #2**

- A map showing the study site has been added into the manuscript

**Reviewer #3**

- The study was undertaken before the 2018 RVF outbreak in Wajir and Marsabit counties.

**Summary and general comments**

**Reviewer #1: No comment**

**Reviewer #2:**

- The practical strategies on how to integrate the knowledge of the nomads into an early warning system have been added and discussed in the document as already detailed in the above sections.

**Reviewer #3**

- Demographics of respondents section and table have been added into the manuscript as already captured in sections above.
- Public health recommendations/strategies on how to integrate the knowledge of the pastoralists into the early warning surveillance system have been elaborated as already detailed in the sections above.

Thank you.

Sincerely,

Dr. Geoffrey Otieno Muga, PHD

Corresponding and First Author
