# Supplementary material for: Indigenous knowledge of Rift Valley Fever among Somali nomadic pastoralists and its implications on public health delivery approaches in Ijara sub-County, North Eastern Kenya
Source: PLoS Negl Trop Dis. 2021 Feb 22;15(2):e0009166. doi: 10.1371/journal.pntd.0009166 (PMC7932528; doi:10.1371/journal.pntd.0009166)
Supplement: Attachment — Submitted filename: PNTD-D-19_02196_R1 Review 2 Comments.docx [file pntd.0009166.s003.docx]

**Review of the Manuscript Number PNTD-D-19-02196**

**Introduction**

The manuscript titled ***indigenous knowledge of Rift Valley Fever among Somali nomadic pastoralists and its implication on public health delivery approaches in Ijara Division, North Eastern Kenya*** presents important anthropological information that can be utilized by authorities immediately before and during outbreaks of Rift Valley Fever (RVF). Specifically, the manuscript mentions aspects of disease knowledge from an *emic* perspective including perceived risk factors associated with the disease including heavy rainfall, swarms of mosquitoes, biting flies and contact with wild animals. Symptoms including froth from nose, bloody diarrhoea, fever, abortions and salivation are also mentioned. The manuscript further points out risk pathways associated with RVF including mosquito bites, drinking raw animal milk and/or blood, infected livestock meat and infected wild animals. The authors postulate that the above anthropological knowledge may be useful in the development of early warning system and the general disease control mechanisms when outbreaks occur.

The information is important to officers working in the domain of public health and medical anthropology in general.

However, there are sections that the authors need to review before publication. These include:

**Author’s Summary**

The authors in the last sentence of the summary indicate that there is need to empower cattle keepers… They, however, need to take note that in Ijara district, the livestock mainly kept include cattle, sheep, goats and donkey. They need to indicate “Livestock keepers” instead of restricting the recommendation to “Cattle keepers”.

**Introduction**

The authors only mention cattle, sheep and goats (page 5) as the livestock species affected by RVF. However, they need to take note that camels are also affected (See WHO 2018 - who.int/ news- room/fact-sheets/detail/rift- valley-fever).

**Methods**

i. The authors need to maintain consistency and correctness in presentation of sub-County. The correct presentation should be sub-County but not Sub County (pg 9) or sub- county (pg 10).
ii. The sentence starting with - ‘Approximately 11,474………’- is not clear. The authors need to specify what the figures stand for. The whole sentence needs review to communicate what the authors desire.
iii. The authors should give all the wards in the sub-County to correctly situate Ijara ward and its characteristics.
iv. On page 11 sentence beginning with ‘Tthe information collected from survey…………..’ ­­­------need review to give out the desired meaning.
v. The unit of analysis (Pg 11) ought to be clarified. It appears the household was the unit of analysis as opposed to “individual herd owner.” This is because the authors indicate that they also focused their study on herders, women and owners.
vi. The research site is not fully presented. Whereas Gedilum sub-location is presented, a reader might want to know the location, how many sub locations and the locations in Ijara sub-County.

**Sample Size Determination**

vii. The Population size (10,000) used in the formula is not explained.

**Results and Discussion**

viii. Pg 18 paragraph 1, should be male gender but not men gender
ix. Presentation of variables in tables (pg 18 and 19), the authors should consider populating the variable in a single composite table.
x. In verbatim quote on pg 31, the source should be separated from the quote.
xi. Page 33 paragraph 3. There is no reason why SAN DIK is capitalized. Instead it should be written as *san dik* (lower case and italic).
xii. Table on pg 34 showed be correctly numbered table 2. The indicated (N) should be in the table but not located in the title.
xiii. The authors should also note that it is the tradition that the list of abbreviations come early in the manuscript.

END
