# Supplementary material for: Indigenous knowledge of Rift Valley Fever among Somali nomadic pastoralists and its implications on public health delivery approaches in Ijara sub-County, North Eastern Kenya
Source: PLoS Negl Trop Dis. 2021 Feb 22;15(2):e0009166. doi: 10.1371/journal.pntd.0009166 (PMC7932528; doi:10.1371/journal.pntd.0009166)
Supplement: Attachment — Submitted filename: Authors Letter on Responses to Reviewers_September 28_2020.docx [file pntd.0009166.s004.docx]

Dr. Geoffrey Otieno Muga, PhD

Corresponding and First Author

Institute of Anthropology Gender and African Studies

University of Nairobi

P. O. Box 30197

Nairobi

September 30, 2020.

The Editorial Manager

PLOS Neglected Tropical Diseases

Dear Sir/Madam,

**Re: Submission of Revised Manuscript: Indigenous knowledge of Rift Valley Fever among Somali pastoralists and its implications on public health delivery approaches in Ijara sub-County North Eastern Kenya**

I take this opportunity on behalf of the authors to thank you very much for sharing with us reviewers’ comments on our manuscript cited above. We have made great efforts to address all the comments by the 3 reviewers. We found the comments very useful and they have made us to greatly enrich the manuscript and make it more comprehensive. Find below for your review our responses to the reviewers’ comments and a description of changes made in each and every section.

**Section: Authors’ Summary**

**Reviewer # 1: No comments on the section**

**Reviewer # 2**

- The term “Livestock keepers” has been used to replace “cattle keepers” as suggested.

**Reviewer # 3: No comment on the section**

**Section: Introduction**

**Reviewer # 1: No comment on the section**

**Reviewer # 2**

- Camels have been added into the list of animals affected by RVF on page 5

**Reviewer # 3: No comment on the section**

**Section: Methods**

**Reviewer # 1: No comment on the section**

**Reviewer # 2:**

- The authors have maintained consistency and correctness in presentation of “sub-County” throughout the document e.g. pages 7,8, 9 and others.
- The sentence starting with “Approximately 11,474……” has been rephrased by adding “people” for clear communication.
- The other wards found in Ijara sub-County namely Ijara, Hulugho and Masalani have been indicated to correctly situate the sub-County.
- The sentence beginning with ‘The information collected from survey………’ under study design section has been rephrased by adding ‘technique’ after ‘survey’for more clarity.
- The study population and unit of analysis have been corrected by stating that the study population comprised of herd owners in Ijara sub-County. The unit of analysis has also been specified as the individual herd owner.
- The study location/site has been fully presented. Apart from Gedeluun sub-location, the authors have given other sub-locations in Sangailu ward as Handaro, Matarba, Sangailu and Wakabharey. All the wards forming Ijara-sub-County have also been presented.
- The population size (10,000) used in the sample size determination has been explained as the population of herd owners.

**Reviewer # 3:**

- Secondary data sources that were consulted have been included as books, journal publication, policy documents and reports from conferences and relevant government departments.
- Explanations on how cleaning and coding of the data into running themes was done have been provided. It has also been stated that all the authors reviewed and validated the coding and running themes for accuracy of data before embarking on data analysis and writing.
- The statement on removable disks and hard copy files has been deleted as recommended.

**Section: Results and Discussion**

**Reviewer #1: No comment on the section**

**Reviewer # 2:**

- Male gender has been used in the 4^th^ sentence under demographics of respondents.
- Table 1 on Socio-demographics of respondents has been redone and variables presented in a single composite table. Frequencies have also been removed as recommended.
- The source of the verbatim quote under RVF healing practices has been separated from the quote.
- Under section on RVF incubation period and severity,  *SANDIK* has been correctly spelt *as san dik* (lower case and in italics)
- Table 2 on gender role matrix has been correctly numbered. The indicated (N) has been deleted.
- The list of abbreviations has been moved to its rightful place just before the introduction section.

**Reviewer # 3:**

- Table 1 on socio-demographics of respondents has been condensed. ‘Total’ under every variable and variable frequencies have been removed. Only three columns have been maintained (variable, category and n (%). Income unit has been defined as Kshs.
- All figures have been clearly labelled to show what is being displayed. E.g. Fig 2 has been indicated as RVF signs and symptoms in animals. Fig 3 has been elaborated as risks and early warning events associated with RVF occurrence and Fig 4 has been indicated as RVF risk pathways into humans and animals.
- On consolidating all survey data in the manuscript into one table, we find the current presentation more sufficient and logical to follow by the reader.

**Section: Conclusion**

**Reviewer# 1: No comment on the section**

**Reviewer # 2: No comment on the section**

**Reviewer #3:**

- The recommendation on the use of mobile phones in RVF control has been supported by including survey data on mobile phone and radio ownership in the socio-demographic table. This shows that the herders have access to these gadgets and can use them to report cases of RVF occurrence. The recommendations have also been indicated as coming from qualitative interviews with key informants (see section under socio-demographics of respondents).
- Some elements on the strengths of the study have been brought to the conclusion section as recommended. Limitation section has also been reviewed and made clearer.

**Section: Abstract**

**Reviewer # 1: No comment**

**Reviewer #2: No comment**

**Reviewer #3:**

- The authors have specified in the abstract how many people were surveyed or participated in the interviews and FGDs. These are survey (204); KIIs (15) and 4 FGDs.

**Other comments**

- A statement on 2018 RVF outbreak has been added in the introduction section.
- The authors have reviewed and corrected notable grammatical errors in the manuscript.

Thank you.

Sincerely,

Dr. Geoffrey Otieno Muga, PHD

Corresponding and First Author
