# Supplementary material for: Indigenous knowledge of Rift Valley Fever among Somali nomadic pastoralists and its implications on public health delivery approaches in Ijara sub-County, North Eastern Kenya
Source: PLoS Negl Trop Dis. 2021 Feb 22;15(2):e0009166. doi: 10.1371/journal.pntd.0009166 (PMC7932528; doi:10.1371/journal.pntd.0009166)
Supplement: Attachment — Submitted filename: Letter of Response to ReviewersComments.docx [file pntd.0009166.s005.docx]

Dr. Geoffrey Otieno Muga, PhD

Corresponding and First Author

Institute of Anthropology Gender and African Studies

University of Nairobi

P. O. Box 30197

Nairobi

January 11, 2021.

The Editorial Manager

PLOS Neglected Tropical Diseases

Dear Sir/Madam,

**Re: Submission of Revised Manuscript: Indigenous knowledge of Rift Valley Fever among Somali pastoralists and its implications on public health delivery approaches in Ijara sub-County North Eastern Kenya**

I take this opportunity on behalf of the authors to thank you very much for sharing with us reviewers’ comments on our manuscript cited above. We have made great efforts to address all the comments by the 3 reviewers. We found the comments very useful and they have made us to immensely enrich the manuscript and make it more comprehensive. Find below for your review our latest responses to reviewer # 3 comments and a description of changes made in each and every section.

**Reviewer 3 Responses to Questions**

**METHOD**

Reviewer #3: The authors adequately addressed the prior recommendations. The secondary data review and interview transcription, coding, and validation methods are all more clearly defined.
 No comment requiring authors’ attention
--------------------

**RESULTS**

Reviewer #3: Table 1 is more concise now that a separate row for total has been removed for each variable. However, it is best practive for the the table to still specify either the denominator (N=204) in the header/title or the numerator should be listed next to each percentage (e.g., Sex        Male     161(79.4%).

Authors’ response: The Denominator (N=204) has been specified in the header/title of the table 1.

The figures 3 and 4 are now labeled and the presented content is more evident. The authors should also consider not rounding to the 100th decimal point- whole number or 10th is sufficient for the percentages presented. As in table 1, neither the numerator or denominator are presented. The denominator (N=204) could be presented in the title, footnote, or consider presenting the numerator (%) above each bar.

Authors’ response: The percentages in figures 2, 3, & 4 have been rounded off to the 10^th^ decimal point as recommended.

Authors’ response: The Denominator (N=204) for figure 2, 3, & 4 has been captured in each figure title.

--------------------
CONCLUSIONS


Reviewer #3: The expansion on mobile phone uptake and implications for notification is better emphasized. Likewise, the conclusion section provides a better summary and interpretation of the findings now.

No comments requiring attention of the authors.

--------------------

<b>Editorial and Data Presentation Modifications?</b></br><br/>
Use  this section for editorial suggestions as well as relatively minor modifications of existing data that would enhance clarity. If the only modifications needed are minor and/or editorial, you may wish to recommend “Minor Revision” or “Accept”.

Reviewer #3: (No Response)

--------------------

Thank you.

Sincerely,

Dr. Geoffrey Otieno Muga, PHD

Corresponding and First Author
